# Improvement of somatic embryogenesis and plantlet conversion in *Oplopanax elatus*, an endangered medicinal woody plant

**DOI:** 10.1186/2193-1801-2-428

**Published:** 2013-08-31

**Authors:** Heung-Kyu Moon, Yong-Wook Kim, Yong-Pyo Hong, So-Young Park

**Affiliations:** Division of Forest Biotechnology, Korea Forest Research Institute, Suwon, 441-847 Republic of Korea; Division of Forest Genetic Resources, Korea Forest Research Institute, Suwon, 441-847 Republic of Korea; Department of Horticultural Science, Chungbuk National University, Cheongju, 361-763 Republic of Korea

**Keywords:** Micropropagation, *Oplopanax elatus*, Conservation, Somatic embryogenesis, Regeneration

## Abstract

*Oplopanax elatus* is a medicinal plant on the verge of extinction because of overexploitation. In the present study, the effects of various factors on enhancing somatic embryogenesis and plantlet conversion were studied. Mature seeds were collected from a total of 13 plants from 4 mountains in South Korea, and the genetic distances were calculated to analyze the effect of genotype on somatic embryogenesis. Results of cluster analysis and the unweighted-pair-group method with arithmetic mean of 13 genotypes indicated the presence of 3 main groups. Both genotype and explant type affected the induction of somatic embryos (SEs). Sorak 2 and root were found to be the most suitable genotype and explant type, respectively, for SE induction in *O. elatus*. Among the different types of carbon sources tested, 5% sucrose induced the maximum number of SEs. The formation and development of SEs were significantly influenced by culture density; thus, 10 mg embryonic callus was found to be the most suitable for SE induction. The highest rates of germination and SE conversion were obtained in a germination medium containing 1.8 gelrite and 3.2 g·l^-1^ agar. In addition, 80% of the plantlets that were transplanted into artificial soil acclimatized successfully. Thus, our results showed that the percentage survival of *O. elatus* during in vitro proliferation could be increased by optimizing to the somatic embryogenesis system.

## Background

*Oplopanax elatus* is a valuable medicinal plant and one of the first Far-Eastern plants to be recommended for research as a source of herbal preparations, similar to ginseng. Because of its highly valued medicinal properties against conditions such as asthenia, depressive states, and hypertension, this rare and endangered species has been historically overexploited throughout Asia, and its demand is increasing (Lee et al. 2002).

The distribution of *O. elatus* in South Korea is considerably limited, and it only occurs in restricted areas on high mountains (Lee et al. 2002). Conventional methods for the vegetative propagation of *O. elatus*, such as plant division, layering, and cutting, have not been effective (Fu and Jin 1992). Moreover, the distribution of weak, less viable zygotic embryos of this species is restricted to high elevations (Lee et al. 2002). Therefore, alternative methods for the rapid multiplication of this plant must be developed for its conservation, as well as for medicinal purposes.

*In vitro* culture has always been a preferred, alternative approach for replicating genetically identical plantlets of rare and endangered species (Kowalski and Von Staden 2001; Negash 2002; Gomes et al. 2003; Martin 2003a, b). Although callus induction and somatic embryogenesis have been widely explored for Araliaceae, studies on somatic embryogenesis in *O. elatus* are comparatively rare (Moon et al. 2006). For this species, callus induction and somatic embryogenesis have been reported by only Cho et al. (1991); however, the authors could not regenerate plantlets. In 2006, plantlets of this species could be successfully regenerated by somatic embryogenesis in our laboratory (Moon et al. 2006). However, because of the low ratio of induction of somatic embryos (SEs) and plantlet conversion, this method could not be established for the mass propagation of *O. elatus*. Another limitation was that viable seeds of this species cannot be easily collected from natural habitats. Consequently, somatic embryogenesis is dependent on other somatic tissues, such as leaves, stems, and roots. In this paper, we have reported an improved production system for the SEs of *O*. *elatus* by investigating the effects of various factors on plantlet conversion.

## Results

### Cluster analysis and genotype effect

**Figure 1:**
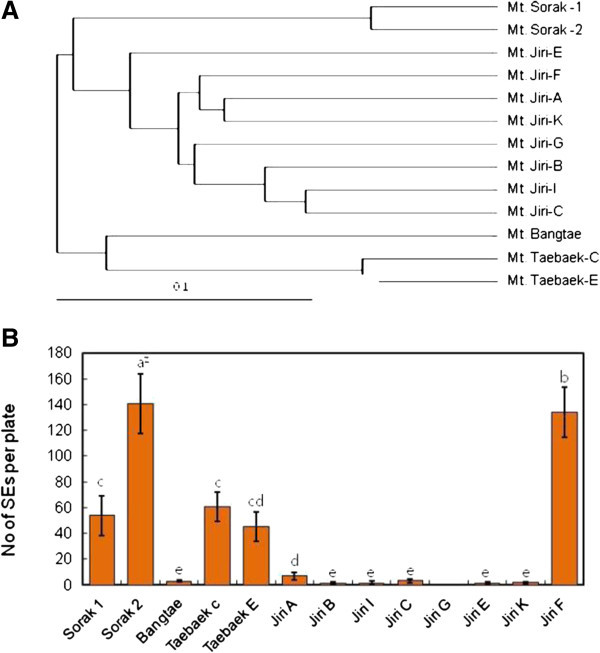
**Genetic distances by UPGMA Cluster analysis. (A)** and genotype effect on somatic embryo (SE) induction **(B)** in *O. elatus*. Vertical bars mean represent mean±standard error of five replicates. ^Z^ mean values followed by the same letters are not significantly different according to Duncan’s multiple-range test at P≤0.05.

The results of the cluster analysis indicated that the genotypes of the studied *O. elatus* specimens can be divided into 3 main clusters (Figure 1A). The Bangtae genotype is related to the 2 Taebaek genotypes, whereas the 2 Sorak genotypes and 8 Jiri genotypes form separate clusters.

Maximum SE formation was observed in Sorak 2 (141.3 ea/plate), followed by Jiri F (136.1 ea/plate). However, the rate of SE formation was low in all the other genotypes from Mt. Jiri (Figure 1B). This result indicates that even the closely related *Oplopanax* genotypes belonging to the same locality vary widely in the ability for SE formation*.*

### SE induction

**Figure 2:**
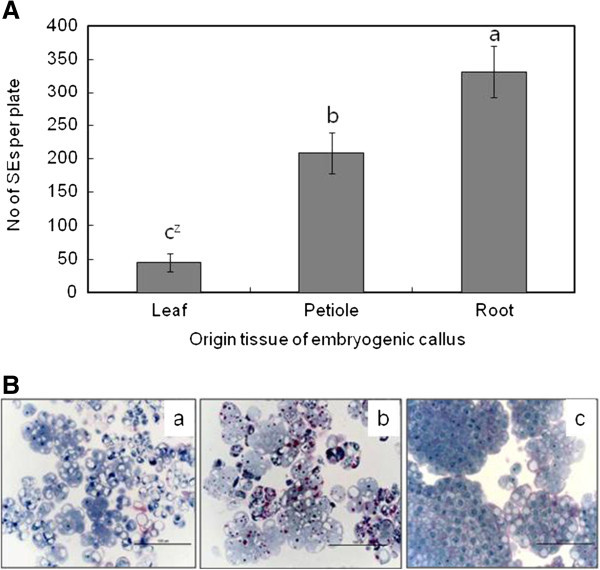
**Effect of origin explants on SEs induction from embryogenic calluses (ECs). A**. Number of SEs induced from different explant-derived ECs, **B**. Histological differences of different explant-derived ECs, a. leaf-derived EC, b. petiole-derived EC, c. root-derived EC. Vertical bars mean represent mean±standard error of five replicates. ^Z^ mean values followed by the same letters are not significantly different according to Duncan’s multiple-range test at P≤0.05.

The type of explant selected also influenced SE induction in *Oplopanax* (Figure 2A). The maximum number of SEs was induced when the root was used as an explant (327.8 ea/plate), followed by the petiole (206.0 ea/plate), and the leaf (49.3 ea/plate). The histological analysis indicated that calli formed from the leaves and stems consisted of vacuolated cells or cell aggregates (Figure 2B-a, b), whereas calli initiated from the roots consisted of small cytoplasmically rich cells that occurred in compact cell clumps (Figure 2B-c).

**Figure 3:**
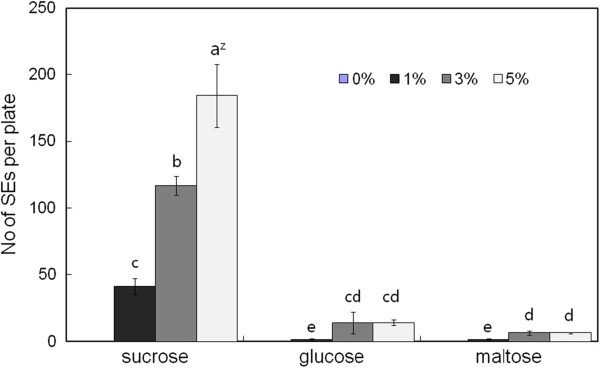
**Effect of carbon sources and concentrations on SE induction in*O. elatus.*** Vertical bars mean represent mean±standard error of five replicates. ^Z^ mean values followed by the same letters are not significantly different according to Duncan’s multiple-range test at P≤0.05.

The type of carbohydrate used as the carbon source was found to influence the number of SEs formed. Sorbitol and mannitol were detrimental to SE induction (data not shown), whereas glucose, maltose, and sucrose favored SE induction when added to the medium (Figure 3). Among these carbohydrates, sucrose was found to be the most suitable carbon source, and the maximum number of SEs (178.6 ea/plate) was formed when 5% sucrose was used compare to lower concentration of sucrose such as 1 (39.2 ea/plate) or 3% (122.4 ea/plate).

**Figure 4:**
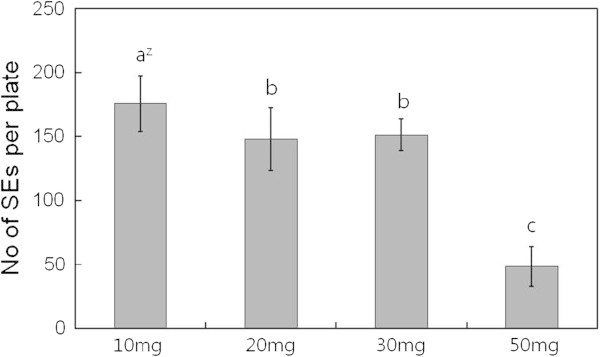
**Effect of cell density on somatic embryo induction from embryogenic callus in*O. elatus*.** Vertical bars mean represent mean±standard error of five replicates. ^Z^ mean values followed by the same letters are not significantly different according to Duncan’s multiple-range test at P≤0.05.

Ten milligrams of embryogenic calli was suitable for SE induction. However, 50 mg of embryogenic calli caused a decline in SE induction (Figure 4) and an increase in EC proliferation instead (data not shown).

**Figure 5:**
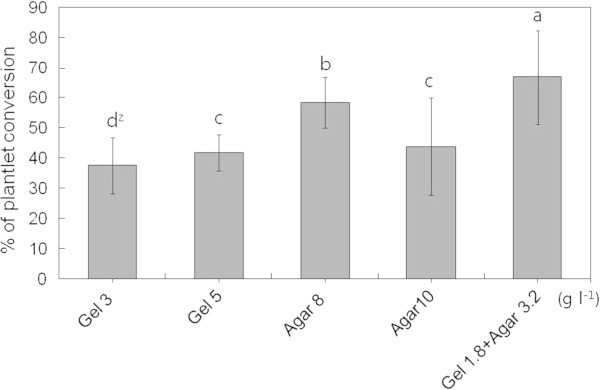
**Effect of gelling agents on SE conversion into plantlets in*O. elatus*.** Vertical bars mean represent mean±standard error of five replicates. ^Z^ mean values followed by the same letters are not significantly different according to Duncan’s multiple-range test at P≤0.05.

Among the gelling agents that were used separately, a high percentage of conversion was also observed when a combination of gelling agents was used, i.e., with 0.18% gelrite and 0.32% agar mixture (Figure 5).

## Discussion

**Figure 6:**
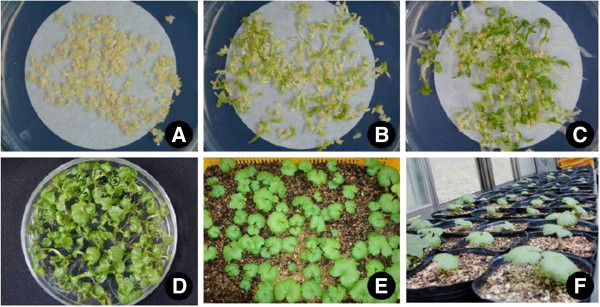
**Plantlets regeneration of*O. elatus*via somatic embryogenesis. A**. Various stages of somatic embryos (SEs) induced on EC induction medium, **B** and **C**. SE maturation and germination on half strength MS medium with 5% sucrose and 0.8% agar, **D**. Converted plantlets on half strength MS medium, **E** and **F**. Acclimatized plantlets in an artificial soil mixture for 5 weeks **(E)** and 2 months **(F)** in greenhouse.

For many rare and endangered plant species, *in vitro* regeneration has been accomplished through organogenesis by using axillary bud and callus cultures (Negash 2002; Gomes et al. 2003; Martin 2003a); however, somatic embryogenesis is not a commonly used method for such species (Augustine and D’ souza 1997; Beena and Martin 2003). In the present study, 200 normally converted young plantlets of *Oplopanax elatus* (about 7 to 10 cm in length) were selected and then transferred to an artificial soil mixture (Figure 6A–F). Eighty percent of the plantlets survived; this led to better survival rates than those observed using our previous protocol (Moon et al. 2006; Park et al. 2011a).

Both the genotype of the plant tissue and the choice of explant type are usually the most important factors in determining the regeneration ability of the plantlets of woody species (Merkle et al. 1997,1998; Chalupa 2000; Bonga 2004). In the present study, a wide variation in SE formation was observed among different genotypes. Similarly, Meurer et al. (2001) observed that in soybean, the genotype had an effect on SE formation during tissue culture. Genetic variability among genotypes may be considered as a factor that influences regeneration capacity during tissue culture (Park et al. 2011b). However, in *O. elatus*, since closely related genotypes show wide variation in SE induction, further studies at the gene level are required to better understand this variation.

In this study, the explant type strongly influenced SE induction in *O. elatus.* Differential responses of different explants from the same source may be achieved by isolating and culturing tissues that are in a more juvenile, potentially regenerative state than most other tissues of a plant (Von Aderkas and Bonga 2000). Further, the callus initiated from the root of *O. elatus* was compact and contained small, cytoplasmically rich embryogenic cells; thus, it formed the so-called “proembryonal complex” which is commonly observed during indirect somatic embryogenesis via callus formation or in “State 1” cell clusters (Zimmerman 1993).

In the present investigation, SE induction was enhanced when the cell density was within a particular range. Somatic embryogenesis is the process in which somatic cells in a culture redifferentiate and give rise to cells that can form SEs, with the occurrence of cell–cell interactions (Mccabe et al. 1997). Cells cultured at low cell densities cannot form SEs; however, cells cultured at a high density, or at a low density in the presence of a cell-free growth medium preconditioned by a high-density suspension culture, can undergo somatic embryogenesis (Smith and Sung 1985; De Vries et al. 1988). However, in this study, higher cell densities of *O. elatus* were unfavorable for somatic embryogenesis, which may be due to competition for nutrients, release of an inhibitor molecule, or non-release of a signal molecule. Further, this discrepancy between our result and those of previous studies may be attributable to fact that the previous studies were conducted using suspension cultures, whereas we used calli.

The exogenous sugar source has been recognized as a major component that influences SE induction (Jain et al. 1995). Ladyman and Girard (1992) observed that among glucose, fructose, sucrose, and maltose, sucrose was the best sugar source for inducing SEs in cucumber. The effect of sucrose on SE induction and maturation has been thoroughly investigated (Tremblay and Tremblay 1995). In spruce, the positive effect of sucrose could not be replaced, even by supplying its monomers, viz., glucose and fructose, to the SE maturation medium (Iraqi and Tremblay 2001). In our study, sucrose resulted in good SE induction at all concentrations, but sugar alcohols could not induce SE formation in *O. elatus*. However, Walker and Parrott (2001) observed that addition of 3% sorbitol increased the germination and conversion frequency of SEs in soybean. Plasmolyzing osmotica, such as sugar alcohols, are known to readily pass through cell walls and cause temporary plasmolysis, until their movement into the cytosol leads to osmotic recovery (Attree and Fowke 1993). This information indicates that in *O. elatus*, sugar alcohols are not suitable for use in SE induction media because of their plasmolyzing nature and, thus, cannot serve as a sugar source.

An important phase in SE development is the process of maturation. During this phase, embryos undergo various morphological and biochemical changes, which are evident from the deposition of storage materials, repression of germination, and acquisition of desiccation tolerance (the latter aspect is mainly observed in species with orthodox seeds) (Blackman et al. 1992; Kermode 1995; Thomas 1993; Mckersie and Brown 1996). In the present study, the effect of gelling agents on SE conversion was investigated. Both the type and concentration of the gelling agent influenced SE conversion in *O. elatus*, with 0.18% gelrite and 0.32% agar combination producing the best results. Similar results were obtained in a previous study in which SE was induced in cucumber (Ladyman and Girard 1992); in this study, agar used separately was found to be better than gelrite. The authors also observed that an increase in agar concentration caused a reduction in the number of SEs. Owens and Wozniac (Owens and Wozniak 1991) investigated the formation of morphogenetic calli from the leaf discs of sugar beet and observed that both gel type and concentration influenced water availability to tissues because of differences in gel matric potential. Further, Pullman et al. (2005) found that during SE development in spruce, the gelling agent strongly influenced ABA adsorption, with agar decreasing the adsorption of ABA to a greater extent than gellan gum (i.e., Gelrite and Phytagel).

The maturation, germination, and conversion of SEs to plants are difficult to attain during somatic embryogenesis (Sutton and Polonenko 1^999^). For woody species in particular, the efficiency of conversion is relatively low, which restricts the application of somatic embryogenesis systems for commercialization purposes (Merkle et al. 1998).

In conclusion, we have developed an improved regeneration system based on somatic embryogenesis for *O*. *elatus*. We expect that by incorporating the changes tested in this study, higher numbers of plantlets of this valuable medicinal woody species will be obtained; this will potentially address the issue of overexploitation of this endangered plant.

## Conclusions

Among various carbohydrates, sucrose was most effective for SE induction, and 5% sucrose induced the maximum number of SEs. The formation of SEs were significantly influenced by culture density, in this study 10 mg/plate embryonic callus was the optimal cell density for SE induction. The highest rates (64%) of conversion were obtained in a germination medium solidified with 1.8 gelrite and 3.2 g·l^-1^ agar.

## Methods

### Plant material and embryogenic callus induction

Mature seeds were collected from total 13 trees at 4 mountains (Mts. Seorak, Jiri, Bangtae and Taebaek), South Korea, in September of 2006, and transported in an icebox to the laboratory. They were surface sterilized with 70% ethanol for 3 min, 2% NaOCl for 30 min, and 0.2% (w/v) HgCl_2_ (Aldrich, USA). The seeds were carefully de-husked with a scalpel. The naked seeds were inoculated on MS medium (Murashige and Skoog 1962) containing l g L^-1^ glutamine, 3% sucrose, 0.3% gelrite (Aidrich, USA), and 1.0 mg L^-1^ 2,4-D for embryogenic callus (EC) induction.

### DNA isolation, simple sequence repeat (SSR) analysis and cluster analysis

Genomic DNA was isolated from embryogenic callus of each genotype following CTAB method. DNA was amplified in a PTC-200 PCR (MJ Research, USA) containing 50 ng template DNA, 1X buffer (75 mM Tris HCl (pH 9.0), 50 mM KCl, 20 mM (NH_4_)_2_SO_4_), 2 mM MgCl_2_, 200 μM of each dNTP, 5 pmol SSR primers, and 1 unit *Taq* DNA polymerase (ABgene, UK). Initial denaturation was at 94°C for 5 min, followed by 94°C for 30 sec, 52°C for 30 sec and 72°C for 30 sec. Cycle was repeated 45 times, followed by 60 sec extension at 72°C. The amplified products were separated on 2% agarose gel and detected by EtBr staining. For cluster analysis, the bands appearing without ambiguity were scored as 1 (present) and 0 (absent) for each primer. Genetic relationship of the individuals was analyzed by UPGMA method (Phylip v3.5c; Felsenstein 1993).

### Induction of somatic embryos (SEs)

For induction of somatic embryos from EC, MS medium was supplemented with ABA 0.1 mg L^-1^, 0.02% activated charcoal, 5% sucrose and 0.5% gelrite. ECs were plated on filter paper (diameter 70 mm) following method of Moon et al. (2008). Cultures were maintained in darkness and examined after 4 weeks of culture.

For investigation of various factors on SE induction viz. genotype effect, explant origin, carbon source, inoculation density and gelling agent the experiments were done as follows:

### Genotype effect

For studying the effect of genotype, EC were induced from the 13 different genotypes from the four different mountains as follows: Two from Sorak, one from Bangtae, two from Taebaek and eight genotypes from Mt. Jiri.

### Explant origin

EC were induced from leaf, petiole and root from somatic plantlets which were grown for 4 weeks in MS medium. Size of explants was leaf 0.5 x 0.5 cm, 1~2-mm petiole and 1-cm root segments. Ten explants were cultured on EC induction medium on a petri-dish with five replications.

### Histological observation

Callus obtained from different explants were fixed in a solution containing 2.5% glutaralde hyde and 1.6% paraformaldehyde buffered with 0.05 M phosphate buffer, pH 6.8, for 24 h at 4°C. Samples were dehydrated in an alcohol series and then embedded in Technovit 7100 (Kulzer, Germany) according to Yeung (1999). Serial 3-μm-thick sections were cut on a Reichert-Jung 2040 Autocut rotary microtome. and stained with toluidine blue O (Yeung 1984).

### Effects of carbohydrate sources and concentrations, inoculation density

Mt. Sorak zyotic embryo-derived EC were used for experiment as plant materials. For studying the effect of carbohydrate source, glucose, fructose, maltose, sorbitol and mannitol were added at concentrations of 1%, 3% or 5% to the medium.

For studying the effect of inoculation density, embryogenic cells were plated following method of Moon et al. (2008). Approximately ten mg of embryogenic calli were plated per plate in 90-mm-diameter petri-dishes.

### Germination and plantlet conversion

Somatic embryos in the early cotyledonary stages were selected and used for these experiments. To improve conversion rate of plantlets from somatic embryos, gelling agents were tested. Half concentration of MS medium containing 20 gL-1 sucrose was solidified with five different combinations of gelling agent; 0.3 and 0.5% gelrite, 0.8 and 1.0% agar, 0.18% gelrite+0.32% agar mixture. For the experiment, around 20 SEs were cultured onto 1.5x9-cm petri-dish, which contained 25 mL of germination medium (GM) completed with different concentrations of gelling agents and GA_3_. The cultures were maintained under light condition (16/8 h, day/night), and after 4 weeks, the number of plantlets converted into plantlets were counted.

### Soil transfer and acclimatization

Two hundred normally converted young plantlets (about 7 to 10 cm long) were selected from the culture dishes and the agar was carefully removed from the roots with tap water. These plantlets were then transferred to an artificial soil mixture (1:2 = PKS2:pelrite, by volume) and cultured in a high-humidity greenhouse to determine their survival after two months.

### Statistical analysis

Experiments were carried out in a randomized design and repeated twice with each treatment having five replicates. The results were subjected to analysis of variance (ANOVA) using the SAS program (SAS Institute, Inc., Cary, NC). Mean values were presented with SE values or separated according to Duncan’s multiple range test at P≤0.05 level.
